# Infections in children with down syndrome and acute myeloid leukemia: a report from the Canadian infections in AML research group

**DOI:** 10.1186/1750-9378-8-47

**Published:** 2013-12-02

**Authors:** Thai Hoa Tran, David Mitchell, David Dix, Sonia Cellot, Marie-Chantal Ethier, Biljana Gillmeister, Johann Hitzler, Victor Lewis, Rochelle Yanofsky, Donna L Johnston, Carol Portwine, Victoria Price, Shayna Zelcer, Mariana Silva, Bruno Michon, Lynette Bowes, Kent Stobart, Josee Brossard, Joseph Beyene, Lillian Sung

**Affiliations:** 1 Division of Haematology/Oncology, The Hospital for Sick Children, 555 University Avenue, Toronto, ON M5G 1X8, Canada; 2 Hematology/Oncology, Montreal Children’s Hospital, Montreal, Canada; 3 Pediatric Hematology/Oncology, British Columbia Children's Hospital, British Columbia, Canada; 4 Hematology/Oncology, Hopital Sainte-Justine, Montreal, Canada; 5 Child Health Evaluative Sciences, The Hospital for Sick Children, Toronto, Canada; 6 Hematology/Oncology/Transplant Program, Alberta Children's Hospital, Alberta, Canada; 7 Hematology/Oncology, Cancer Care Manitoba, Manitoba, Canada; 8 Division of Hematology/Oncology, Children's Hospital of Eastern Ontario, Toronto, Canada; 9 Hematology/Oncology, McMaster Children’s Hospital, Hamilton, Canada; 10 Pediatrics, IWK Health Centre, Halifax, Canada; 11 Hematology/Oncology, London Health Sciences Centre, London, UK; 12 Hematology/Oncology, Cancer Centre of Southeastern Ontario at Kingston, Kingston, Canada; 13 Pediatric Hematology/Oncology, Centre Hospitalier Universitaire de Quebec, Quebec, Canada; 14 Hematology/Oncology, Janeway Child Health Centre, St John, Canada; 15 Stollery Children's Hospital, University of Alberta Hospital, Alberta, Canada; 16 Hematology/Oncology, Centre Hospitalier Universitaire de Sherbrooke, Sherbrooke, Canada; 17 Population Genomics Program, Department of Clinical Epidemiology and Biostatistics, McMaster University, Hamilton, Canada

**Keywords:** Down syndrome, Acute myeloid leukemia, Infection, Chemotherapy, Children

## Abstract

**Background:**

Children with Down syndrome (DS) are at high risk of infectious toxicity when treated with acute lymphoblastic leukemia chemotherapy protocols optimized in children without DS. Our objective was to determine if children with DS and acute myeloid leukemia (AML) have a different risk of infection when treated with chemotherapy protocols developed for children with DS compared to AML treatment protocols developed for children without DS.

**Methods:**

We conducted a retrospective, population-based cohort study that included DS children ≤ 18 years of age with *de novo*, non-M3 AML diagnosed between January 1995 and December 2004, and treated at 15 Canadian centers. Patients were monitored for infection from initiation of AML treatment until recovery from the last cycle of chemotherapy, conditioning for hematopoietic stem cell transplantation, relapse, persistent disease or death (whichever occurred first). Trained research associates abstracted all information from each site.

**Results:**

There were 31 children with DS included; median age was 1.7 (range 0.1-11.1) years. Eleven were treated according to a DS-specific protocol while 20 were treated with non-DS specific protocols. A total of 157 courses of chemotherapy were delivered. Microbiologically documented sterile site infection occurred in 11.9% and 14.3% of DS-specific and non-DS specific AML treatment courses respectively. Sepsis was rare and there were no infection-related deaths. In multiple regression, treatment with a DS-specific protocol was independently associated with a reduction in microbiologically documented sterile site infection (adjusted odds ratio (OR) 0.65, 95% confidence interval (CI) 0.42-0.99; P = 0.044), and clinically documented infection (adjusted OR 0.36, 95% CI 0.14-0.91; P = 0.031) but not bacteremia (adjusted OR 0.73, 95% CI 0.44-1.22; P = 0.231).

**Conclusions:**

Our study suggests that children with DS do not experience excessive infectious toxicity during treatment for AML compared to children without DS. Incorporation of DS-specific AML treatment protocols is associated with a more favorable infection profile for children with DS-AML.

## Background

Children with Down syndrome (DS) have an approximately 10-20 fold increased risk of developing leukemia compared with non-DS children [1,2]. Consequently, children with DS comprise approximately 3% of pediatric acute lymphoblastic leukemia (ALL) [3] and up to 15% of pediatric acute myeloid leukemia (AML) cases enrolled in clinical trials [4]. Recent studies have provided new insights into the unique epidemiology, pathogenesis and treatment response of ALL and AML in children with DS [2]. AML occurs at a younger age in children with DS [5], is defined by somatic *GATA1* mutations [6] and shows increased sensitivity to chemotherapeutic agents such as cytarabine and anthracyclines [7,8].

Excessive mortality of children with DS has been observed during treatment for ALL. These deaths have primarily been attributed to infection after chemotherapy-induced myelosuppression during induction as well as post-remission therapy [9-12]. These observations have led to treatment modifications specifically for DS-ALL. In contrast to the known risk of infection-related mortality for children with DS undergoing ALL treatment, there is a paucity of data defining the risk of infection among children with DS undergoing AML therapy, either with DS-specific or standard AML treatment protocols. The objective of our study was to determine if children with DS-AML have a lower risk of infection when treated with DS-specific AML chemotherapy protocols compared to standard AML treatment protocols developed for children without DS.

## Methods

In this retrospective, population-based cohort study, we included children with newly diagnosed AML who had a diagnosis of DS and were treated at one of 15 Canadian centers that care for children with cancer in each province except for Saskatchewan. This manuscript is a follow-up analysis of a larger study in which outcomes of all children with newly diagnosed AML in Canada were analyzed [13].

### Study sample

We included children with DS (age ≤ 18 years) who were diagnosed with AML between January 1, 1995 and December 31, 2004. We excluded those with acute promyelocytic leukemia, secondary AML and previous diagnosis of immunodeficiency. We collected information on infections from the start of AML treatment until hematopoietic recovery from the last cycle of chemotherapy, start of conditioning for hematopoietic stem cell transplantation (HSCT), relapse, change in protocol therapy due to refractory disease, or death (whichever occurred first). Trained clinical research associates abstracted and coded the relevant information (see below). Intensive chemotherapy was defined as treatment expected to result in profound neutropenia.

### Outcome measures

We described the occurrence of microbiologically documented sterile site infection [14], bacteremia and clinically documented infection during each course of chemotherapy. Sterile site cultures with common contaminants such as coagulase-negative Staphylococcus were only considered true infection if there were two or more positive cultures within the same episode or if the infection was associated with sepsis [15,16]. Sepsis was defined as systemic inflammatory response syndrome in the presence of suspected or proven infection and organ dysfunction according to international consensus guidelines [17,18]. We classified clinically documented infections based upon the Centers for Disease Control and Prevention (CDC) definitions of nosocomial infections [19]. Bacteremia was included as a sterile site microbiologically documented infection such that any true bacteremia would be counted in both outcomes, but would not be included as a clinically documented infection unless associated with a specific site of infection such as pneumonia or cellulitis.

### Potential predictors

We described demographic and course characteristics according to whether the child was treated with a DS-specific or a non-DS specific AML treatment protocol. A DS-specific AML protocol was defined as a treatment protocol used only for children with DS and not a treatment protocol that included both children with and without DS. In addition, the following variables were evaluated to determine if they were potential confounders in the relationship between protocol type and infection outcomes: child age at diagnosis, diagnosis prior to January 1, 2000, cumulative dose of cytarabine in grams/m^2^, severe neutropenia defined as an absolute neutrophil count (ANC) ≤ 500/uL at the start of the course, neutropenia >15 days (threshold chosen a priori), and number of days during which systemic corticosteroids were administered for any reason.

### Statistics

In order to compare demographic features of patients treated with DS and non-DS specific protocols, continuous variables were compared using the Wilcoxon rank sum test and categorical variables were compared using the Chi square or Fisher’s exact test as appropriate. Course characteristics were not statistically compared since courses were not independent within an individual child. Factors associated with the occurrence of microbiologically documented sterile site infection, bacteremia and clinically documented infection were examined using a repeated measures logistic regression with generalized estimating equations and the association was expressed as an odds ratio (OR) with 95% confidence interval (CI). To determine if treatment with a DS-specific protocol was independently associated with infection outcomes, variables significant in univariate analysis were added to the multiple regression model that contained treatment protocol type. Spearman correlation coefficients were evaluated to ensure lack of co-linearity before addition to the model. All tests of significance were two-sided, and statistical significance was defined as P <0.05. Statistical analysis was performed using the SAS statistical program (SAS-PC, version 9.3; SAS Institute Inc., Cary, NC).

### Ethical approvals

This study was approved by the Research Ethics Board at The Hospital for Sick Children and local Research Ethics Boards of the 14 other participating sites (McMaster University-Hamilton Health Sciences/Faculty of Health Sciences Research Ethics Board, Montreal Children’s Hospital Research Ethics Board, Children’s Hospital of Eastern Ontario Research Ethics Board, University of Winnipeg Research Ethics Board, University of British Columbia/Children’s and Women’s Health Centre of British Columbia Research Ethics Board, Centre Hospitalier Universitaire Sainte-Justine Research Ethics Board, University of Calgary Conjoint Health Research Ethics Board, IWK Research Ethics Board, Queen’s University-Health Sciences Research Ethics Board, University of Western Ontario Research Ethics Board for Health Science Research Involving Human Subjects, Memorial University Human Investigation Committee, Centre Hospitalier Universitaire de Quebec Research Ethics Board, University of Alberta Health Research Ethics Board-Biomedical Panel, Centre Hospitalier Universitaire de Sherbrooke Research Ethics Board). As this was a retrospective review study the Research Ethics Board at The Hospital for Sick Children and those at the 14 other participating sites waived the need for written informed consent.

## Results

The primary AML study included 341 patients; 168 (49.3%) were male and the median age was 7.1 (interquartile range 2 to 13.5) years [13]. Thirty-one children with DS were included in the current analysis; the median age was 1.7 (range 0.1 to 11.1) years. Eleven were treated with DS-specific AML protocols while 20 were treated with non-DS specific AML protocols. The DS-specific AML protocols were: Children’s Oncology Group (COG) A2971 (n = 9) and the AMKL-DS low dose cytarabine protocol (n = 2). The non-DS specific protocols were: Pediatric Oncology Group (POG) 8821 (n = 1), CCG 213 (n = 2), CCG 2891 (n = 4), and POG 9421 (n = 13). Characteristics including age and time on intensive chemotherapy were not significantly different between those treated with DS-specific versus non-DS specific AML protocols (Table 1).

**Table 1:** Characteristics of children with Down syndrome at diagnosis of acute myeloid leukemia (N = 31)

	**DS-specific AML protocol (N = 11)**	**Non-DS specific AML protocol (N = 20)**	**P value**
**Child characteristics at diagnosis**			
Male (%)	9 (81.8)	11 (55.0)	0.241
Median age in years (IQR)	1.5 (0.8, 2.3)	2.2 (1.5, 2.9)	0.132
Median WBC (×10^9^/L)(IQR)	5.4 (3.1, 21.1)	7.6 (4.8, 16.7)	0.397
Median ANC (×10^9^/L)(IQR)^a^	1.1 (0.6, 2.6)	1.6 (0.8, 4.1)	0.312
Median hemoglobin (g/L)(IQR)	82.0 (78.0, 107.0)	92.5 (59.0, 110.0)	0.870
Median platelet count (×10^9^/L )(IQR)^b^	33.5 (12.0, 57.0)	44.0 (30.0, 77.0)	0.261
Cytogenetics (%)			0.392
Normal karyotype	2 (18.2)	1 (5.0)	
t(8;21), inv16 or t(16;16)	0 (0.0)	1 (5.0)	
11q23 abnormalities	0 (0.0)	2 (10.0)	
Unknown	3 (27.3)	6 (30.0)	
Other	6 (54.5)	10 (50.0)	
**Treatment characteristics**			
Registered on a study (%)	2 (18.2)	6 (30.0)	0.676
Time on any chemotherapy (median days) (IQR)	199.0 (177.0, 260.0)	168.5 (136.5, 225.0)	0.143
Time on intensive chemotherapy (median days) (IQR)	135.0 (120.0, 143.0)	153.5 (129.0, 214.0)	0.094

*Abbreviations*: *IQR* interquartile range, *WBC* white blood cell, *ANC* absolute neutrophil count, *AML* acute myeloid leukemia, *DS* Down syndrome.

^a^ANC not available at diagnosis for two patients in the non-Down syndrome protocol group.

^b^Platelet count not available at diagnosis for one patient in both groups.

The characteristics of 157 courses of AML chemotherapy observed for this analysis are shown in Table 2. Microbiologically documented sterile site infection occurred in 11.9% of DS-specific and 14.3% of non-DS specific AML treatment courses. Sepsis was rare and there were no infection-related deaths.

**Table 2:** Course characteristics and infection outcomes according to acute myeloid leukemia protocol type (N = 157)

	**DS-specific AML protocol (N = 59)**	**Non-DS AML protocol (N = 98)**
**Course characteristics**		
Number with neutropenia (ANC <0.5 x10^9^) at start of course (%)	5 (8.4)	12 (12.5)^a^
Median days with neutropenia (IQR)	11.0 (3.0, 21.0)	16.0 (7.0, 25.0)
Median days receiving steroids (IQR)	0.0 (0.0, 2.0)	2.0 (0.0, 5.0)
**Supportive care**		
Co-trimoxazole prophylaxis (%)	41 (69.5)	66 (67.4)
Fluconazole prophylaxis (%)	28 (47.5)	39 (39.8)
**Infection outcomes**^**b**^		
Microbiologically documented sterile site infection (%)	7 (11.9)	14 (14.3)
Bacteremia (%)	6 (10.2)	10 (10.2)
Sterile site Gram-positive bacteria (%)	5 (8.5)	9 (9.2)
Sterile site Gram-negative bacteria (%)	2 (3.4)	6 (6.1)
Sterile site fungus (%)	0 (0.0)	1 (1.0)
Clinically documented infection (%)	7 (11.9)	29 (29.6)
Sepsis (%)	1 (1.7)	3 (3.1)
Infectious death (%)	0 (0.0)	0 (0.0)

*Abbreviations*: *ANC* absolute neutrophil count, *IQR* interquartile range, *AML* acute myeloid leukemia, *DS* Down syndrome.

^a^ANC not available at start of 2 courses.

^b^Infection outcomes represent at least one event per course.

We then evaluated factors potentially associated with the occurrence of microbiologically documented sterile site infection, bacteremia and clinically documented infection (Table 3). Treatment with a DS-specific AML protocol was significantly associated with a reduction in these infection outcomes. In multiple regression, treatment with a DS-specific protocol was independently associated with a reduction in microbiologically documented sterile site infection (adjusted OR 0.65, 95% CI 0.42 to 0.99; P = 0.044), and clinically documented infection (adjusted OR 0.36, 95% CI 0.14 to 0.91; P = 0.031) but not bacteremia (adjusted OR 0.73, 95% CI 0.44 to 1.22; P = 0.231).

**Table 3:** Factors associated with infection outcomes per course of chemotherapy (N = 157)

	**Microbiologically documented sterile site infection**		**Bacteremia**		**Clinically documented infection**	
	**Odds ratio (CI)**	**P value**	**Odds ratio (CI)**	**P value**	**Odds ratio (CI)**	**P value**
Down syndrome-specific treatment protocol	0.42 (0.28, 0.64)	<0.0001	0.48 (0.29, 0.78)	0.003	0.25 (0.10, 0.60)	0.002
Age in years	1.01 (0.99, 1.03)	0.349	1.01 (0.98, 1.03)	0.577	1.01 (0.99, 1.03)	0.261
Diagnosed prior to January 1, 2000	0.79 (0.60, 1.03)	0.080	0.75 (0.56, 1.00)	0.052	0.95 (0.75, 1.22)	0.703
Cumulative dose of cytarabine (g/m^2^)	1.03 (1.02, 1.05)	0.0001	1.03 (1.01, 1.05)	0.001	0.99 (0.97, 1.01)	0.184
Neutropenia (ANC <0.5 ×10^9^) at start of course	1.01 (0.72, 1.43)	0.936	0.82 (0.56, 1.22)	0.336	1.88 (1.38, 2.56)	<0.0001
Greater than 15 days with neutropenia	2.50 (1.89, 3.32)	<0.0001	2.45 (1.83, 3.36)	<0.0001	2.81 (2.16, 3.66)	<0.0001
Days receiving steroids	1.08 (1.06, 1.11)	<0.0001	1.07 (1.05, 1.10)	<0.0001	1.07 (1.05, 1.09)	<0.0001

*Abbreviations*: *ANC* absolute neutrophil count, *CI* confidence interval.

The organisms underlying the infections observed in children with DS treated with both DS-specific and non-DS specific AML protocols are shown in Table 4.

**Table 4:** Microbiologically documented infection observed during acute myeloid leukemia therapy in children with Down syndrome

	**DS-specific AML protocol (N = 12)**	**Non-DS specific AML protocol (N = 38)**
Sterile site bacteria*		
Gram positive		
Viridans group streptococci	5	4
Coagulase negative staphylococci	0	3
*Enterococcus faecalis*	0	2
Other^a^	0	1
Gram negative		
*Escherichia coli*	1	3
*Klebsiella pneumoniae*	0	1
*Enterobacter cloacae*	0	2
Others^b^	1	2
Fungus*		
*Candida species*	0	2
Virus*		
Herpes simplex virus	1	1
Respiratory syncytial virus	1	3
Torovirus	1	4
Others^c^	1	5
Clostridium difficile*	1	5

*Abbreviations*: *AML* acute myeloid leukemia, *DS* Down syndrome.

*For bacterial infections other than C. difficile, only sterile site positive cultures are shown. For fungi, viruses and C. difficile, both sterile and non-sterile site positive cultures are shown.

^a^Other: beta-hemolytic *Streptococcus* (n = 1).

^b^Others: Citrobacter freundii (n = 1) for Down syndrome protocol group; *Enterobacter species* (n = 1) and Haemophilus influenza (n = 1) for non-Down syndrome protocol group.

^c^Other: parvovirus (n = 1) for Down syndrome protocol group; parainfluenza (n = 3) and rotavirus (n = 2) for non-Down syndrome protocol group.

## Discussion

In this population-based study of children with DS and AML, we found that microbiologically documented sterile site infection occurred in less than 15% of chemotherapy courses, whether children with DS were treated with a DS-specific or standard AML treatment protocol. Sepsis was rare and there were no infection-related deaths. We found that treatment with a DS-specific AML protocol was associated with fewer microbiologically documented sterile site infection and clinically documented infection in children with DS-AML.

The relatively low risk of infection for children with DS undergoing AML therapy is surprising. More than 60% of non-DS children with AML treated according to the CCG 2961 protocol experienced an infection during each course of chemotherapy [20]. Similarly, in a more recent pediatric AML phase III trial, AAML0531, 30 to 60% of courses were complicated by sterile site infections [21]. While treatment with a DS-specific AML protocol may partially explain the low rate of infection in this cohort, it remains unclear why children with DS treated with non-DS specific AML protocols also experienced a low rate of infection. It is possible that clinicians making decisions regarding treatment modifications or enhanced supportive care for children with DS-AML were influenced by the data about excess infection-related complications in DS-ALL. Another factor to consider is that neither CCG 2961 nor AAML0531 infection reports had the ability to evaluate common contaminants and to distinguish these from true bacteremia; this issue may have artificially increased the infection rates on those studies.

Highly aggressive chemotherapy protocols for AML were associated with excessive mortality in children with DS [5]. In contrast, standard and reduced intensity AML protocols have resulted in superior survival among children with DS compared to non-DS children with AML [22,23], supporting the development of DS-specific AML treatment protocols. The reason why DS-specific AML protocols are associated with fewer infections may be related to the reduced intensity of therapy associated with these protocols. It has been shown that higher cumulative cytarabine dose is associated with higher risk of infection [24,25]. However, infection risk did not correlate with cumulative cytarabine dose in the report by the Japanese Children’s Cancer and Leukemia Study Group (JCCLSG) AML 9805 in which highest infection-related death (12.5%) among children with DS-AML occurred after a cumulative cytarabine dose of 12.6 g/m^2^[26]. In contrast, treatment-related mortality was 5% in BFM98 for children with DS-AML after a cumulative cytarabine dose of 29 g/m^2^ and 5% in NOPHO AML93 with a cumulative cytarabine dose of 48.6 g/m^2^[27,28]. Interestingly, the cumulative dose of cytarabine and anthracycline between DS-specific and non-DS specific AML protocols used in our cohort is overall comparable with the exception of the AMKL-DS low-dose cytarabine regimen and POG 8821 (see Additional file 1). The incorporation of additional chemotherapy agents such as etoposide and dexamethasone and intensive-timing chemotherapy delivery in non-DS specific AML protocols may perhaps account for this difference in infectious toxicities.

Different cooperative groups are considering further reduction of treatment intensity for children with DS-AML in view of toxicity concerns and the unique enhanced sensitivity of DS-AML cells to cytarabine [7,8,29]. However, in light of the low infectious morbidity and mortality in our study, further treatment intensity reduction might not provide additional safety advantages but may compromise event-free survival rates in children with DS-AML. Subsequent trials for DS-AML will need to carefully consider whether further reduction in treatment intensity is warranted and in contrast, may elect to further enhance supportive care in order to further reduce toxic events.

The major strength of our study lies in the fact that this was a population-based study which allowed us to capture all patients with DS-AML treated as opposed to only those treated at a single institution or registered on clinical trials. Therefore, we believe that the present study provides an accurate estimate of the incidence of different infection outcomes within this population. The rigor in identifying infections using a common group of well-trained personnel represents another strength of this study. In addition, each outcome measure was examined at a course level and not a patient level; this approach allowed us to evaluate factors that are expected to change between courses. However, interpretation and generalization of our results require some caution due to the retrospective nature and the small sample size of our study. More specifically, the small number of children included in our study limits the precision of estimates although there is no reason to suspect selection bias may have occurred given the population-based retrospective nature of the study.

## Conclusion

In conclusion, our study suggests that children with DS-AML do not experience excessive infectious toxicity during treatment for AML compared to the general pediatric population without DS. In children with DS, treatment protocols specifically developed for DS-AML were associated with a more favorable profile of infectious toxicity compared to standard AML treatment protocols, supporting the development of specific treatment approaches for this distinct form of pediatric AML.

## Abbreviations

DS: Down syndrome; DS-ALL: DS and acute lymphoblastic leukemia; DS-AML: DS and acute myeloid leukemia; ANC: Absolute neutrophil count; AML: Acute myeloid leukemia; ALL: Acute lymphoblastic leukemia; HSCT: Hematopoetic stem cell transplantation; CDC: Centers for disease control; OR: Odds ratio; CI: Confidence interval; AMKL-DS: Acute megakaryoblastic leukemia and down syndrome; COG: Children’s oncology group; CCG: Children’s cancer group; POG: Pediatric oncology group; JCCLSG: Japanese children’s cancer and leukemia study group; BFM: Berlin-Frankfurt-Munster; NOPHO: Nordic society of pediatric hematology and oncology.

## Competing interests

The authors declare that they have no competing interests.

## Authors’ contributions

THT and LS were responsible for writing the manuscript. All authors were involved in the design of the research. JB and LS were involved in the analysis of the data. All authors performed the research and have critically reviewed and approved the manuscript.

## Supplementary Material

Additional file 1 Comparison of DS-specific and non-DS specific AML chemotherapy regimens. Click here for file

## Canadian Infections in AML Research Group

David Dix (PI), Buffy Menjou (CRA) and Nita Takeuchi (CRA) from British Columbia Children's Hospital; Kent Stobart (PI), Brenda Ennis (CRA) and Linda Churcher (CRA) from Stollery Children's Hospital; Victor Lewis (PI), Janice Hamilton (CRA) and Karen Mazil (CRA) from Alberta Children's Hospital; Sonia Cellot (PI), Dominique Lafreniere (CRA) and Catherine Desjean (CRA) from Hospital Sainte-Justine; Victoria Price (PI), Tina Bocking (CRA), Lynn Russell (CRA) and Emily Murray (CRA) from IWK Health Centre; Lynette Bowes (PI) and Gale Roberts (CRA) from Janeway Child Health Centre; Carol Portwine (PI) and Sabrina Siciliano (CRA) from McMaster Children’s Hospital; Joseph Beyene (Collaborator) from McMaster University; Mariana Silva (PI) from Kingston General Hospital; Rochelle Yanofsky (PI), Rebekah Hiebert (CRA) and Krista Mueller (CRA) from CancerCare Manitoba; Shayna Zelcer (PI), Martha Rolland (CRA) and Julie Nichols (CRA) from London Health Sciences; Donna Johnston (PI) and Elaine Dollard (CRA) from Children’s Hospital of Eastern Ontario; David Mitchell (PI), Martine Nagy (CRA) and Margaret Hin Chan (CRA) from Montreal Children's Hospital; Bruno Michon (PI), Josee Legris (CRA) and Marie-Christine Gagnon (CRA) from Centre Hospitalier Universitaire de Quebec; Josee Brossard (PI) and Lise Bilodeau (CRA) from Centre Hospitalier Universitaire de Sherbrooke; Lillian Sung (PI), Biljana Gillmeister (CRA), Marie-Chantal Ethier (CRA), Renee Freeman (Collaborator), Jeffrey Traubici (Collaborator), and Upton Allen (Collaborator) from The Hospital for Sick Children.
