# Supplementary material for: Infections in children with down syndrome and acute myeloid leukemia: a report from the Canadian infections in AML research group
Source: Infect Agent Cancer. 2013 Dec 2;8:47. doi: 10.1186/1750-9378-8-47 (PMC4174901; doi:10.1186/1750-9378-8-47)
Supplement: Additional file 1 — Comparison of DS-specific and non-DS specific AML chemotherapy regimens. [file 1750-9378-8-47-S1.doc]

**Additional file 1. Comparison of DS-specific and non-DS specific AML chemotherapy regimens**

| Protocols | Daunorubicin (mg/m^2^)* | Cytarabine (g/m^2^)* | Etoposide | Dexamethasone |
| --- | --- | --- | --- | --- |
| **DS-specific** |  |  |  |  |
| COG A2971 | 320 | 27.2 | No | No |
| AMKL-DS | No | 4.8 | No | No |
| **Non-DS specific** |  |  |  |  |
| POG 8821 | 360 | 73.7 | Yes | No |
| POG 9421 | 279-375 | 20.7-34.0 | Yes | No |
| CCG 213 | 180-425 | 27.1 | Yes | Yes |
| CCG 2891 | 320 | 28.2 | Yes | Yes |

Abbreviations: AML – acute myeloid leukemia ; DS – Down syndrome

*Cumulative dose
